# Messages across time and space

**DOI:** 10.7554/eLife.63845

**Published:** 2020-11-17

**Authors:** Sascha M Kuhn, André Nadler

**Affiliations:** Max Planck Institute of Molecular Cell Biology and GeneticsDresdenGermany

**Keywords:** signaling compartmentalization, cAMP, pancreatic beta cell, second messengers, oscillation, homeostasis, Mouse

## Abstract

Compartmentalized oscillations of second messengers affect global cellular signaling.

**Related research article** Tenner B, Getz M, Ross B, Ohadi D, Bohrer CH, Greenwald EC, Mehta S, Xiao J, Rangamani P, Zhang J. 2020. Spatially compartmentalized phase regulation of a Ca^2+^-cAMP-PKA oscillatory circuit. *eLife* **9**:e55013. doi: 10.7554/eLife.55013

Homeostasis – the ability of an organism to maintain a stable internal environment despite changes in the outside world – is essential for cells, tissues and organs to work properly. Regulating body temperature, maintaining blood pressure or monitoring blood sugar levels are all different forms of homeostasis, and they depend on specialized sensory cells. These cells monitor the environment and the body’s condition and, if necessary, chime in to keep the body functioning properly.

Sensory cells are, in turn, tightly regulated by so-called second messengers – small intracellular signaling molecules that control many different pathways within a cell. The β-cells in the pancreas, for example, are responsible for sensing elevated blood sugar levels and for releasing insulin. This hormone regulates how the body uses and stores glucose and fat. If excessive glucose concentrations are detected in the blood, second messenger molecules in the β-cells, such as calcium ions and cyclic adenosine monophosphate (cAMP), are modulated (Komatsu et al., 2013; Rorsman and Ashcroft, 2018). Temporal changes in the concentration of these two molecules cause the secretory granules (compartments within the β-cells that store insulin) to fuse with the plasma membrane, thereby releasing insulin into the blood stream (Rorsman and Ashcroft, 2018; Figure 1).

**Figure 1.**
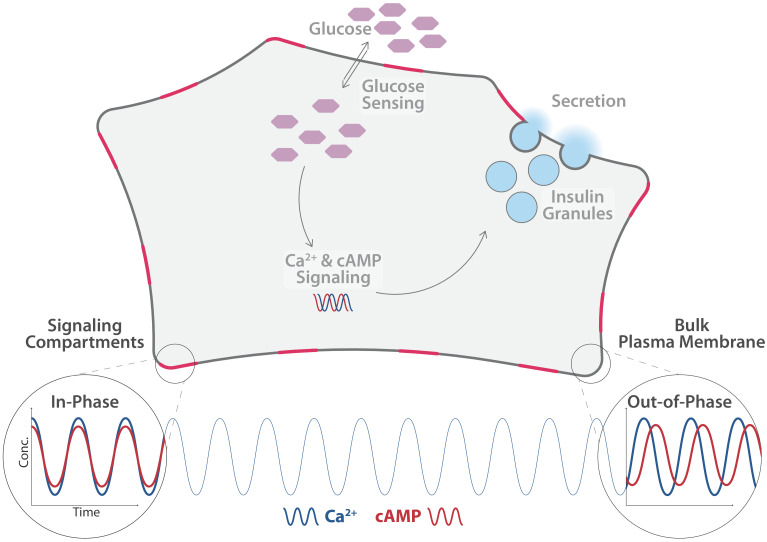
Distinct pools of cyclic adenosine monophosphate (cAMP) exist within individual β-cells. cAMP and calcium ions (Ca^2+^) are important second messengers in cells. In the β-cells of the pancreas, they regulate the release of insulin (light blue) into the blood stream, . Tenner et al. found that the level of Ca^2+^ (blue line) oscillates uniformly across the cell. The level of cAMP (red line) in the cytosol oscillates in phase with the level of Ca^2+^, as does the level in free pools of cAMP in the plasma membrane (bottom right). However, the level in spatially constrained pools of cAMP in the plasma membrane (shown here in pink) oscillates out-of-phase with Ca^2+^ and cAMP in the cytosol in the plasmaand elswhere in the membrane.

In β-cells, the concentrations of calcium ions and cAMP oscillate to integrate external and internal signaling cues and encode them into parameters such as amplitude and frequency (Parekh, 2011; Schöfl et al., 1994). Depending on these parameters, different processes are elicited by the activity of second messengers. Previous research has shown that second messengers are not only controlled at various times, they are also spatially divided within the cells. So far, however, it remains unclear how the spatial organization of these highly diffusible molecules is achieved. Now, in eLife, Jin Zhang and colleagues from the University of California, San Diego – including Brian Tenner as first author – report new insights into second messenger oscillations (Tenner et al., 2020).

Tenner et al. used targeted biosensors to measure cAMP levels in various areas within β-cells. They identified different, spatially separated pools of cAMP, which were set apart by different oscillation patterns and concentrations of cAMP. More specifically, cAMP pools situated near specific protein complexes in the plasma membrane oscillate out-of-phase with cAMP pools located freely in the plasma membrane or in the cytosol. In comparison, calcium ion levels oscillated uniformly in the entire cell.

Using super resolution microscopy, the researchers found that the enzymes that produce and degrade cAMP play an important role in the oscillation process. The enzyme that synthesizes cAMP forms clusters in the plasma membrane, while the enzyme that degrades cAMP is dispersed in the cytosol. Based on these observations, Tenner et al. compiled a mathematical model, which confirmed that the different distributions of the two enzymes enables cAMP to form compartmentalized pools close to the plasma membrane clusters, where it is produced.

An enzyme known as protein kinase A further processes the signals encoded in the second messenger concentrations by helping calcium ions to enter the cell, thereby affecting calcium ion concentrations in the entire cell. Tenner et al. demonstrated that this effect of protein kinase A is dependent on the clustering of enzymes that produce cAMP: when these clusters were disrupted, the oscillations of calcium ions in the cytosol were less sustained and asynchronous. This suggests that modulating the oscillations of second messengers in distinct signaling compartments can affect communication in the entire cell.

While this work adds important new insights into oscillatory second messenger networks, it is based on observations made using an ammonium salt to stimulate a signaling responses. This pharmacological treatment might induce responses inside the cell that could differ from those observed after a physiological stimulation. In the future, it will be important to examine how the compartments of cAMP react to relevant physiological cues such as elevated blood sugar or blood fat levels. It will also be crucial to investigate whether the observed effects on calcium ions in the cell ultimately influence the production of insulin.

Nevertheless, Tenner et al. describe a new mechanism that may change how we think about cell signaling. Oscillatory second messenger networks integrate external and internal signals and are therefore crucial for cellular information processing. Amplitude and frequency of second messenger oscillations have long been established as parameters relevant for encoding informationIt is well known that the amplitude and frequency of these oscillations can encode information (Parekh, 2011; Schöfl et al., 1994). The findings of Tenner et al., however, suggest that the relative oscillatory phase of spatially discrete cAMP pools may constitute an additional mode for encoding information. In such a conceptual framework, a third mode of information processing would significantly increase the cell’s capability to integrate information and respond to changes in its environment (Waltermann and Klipp, 2011).
